# Supplementary material for: Reasoning with programs in replay
Source: bioRxiv. 2025 Oct 13:2025.10.10.681671. Preprint. [Version 1] doi: 10.1101/2025.10.10.681671 (PMC12632884; doi:10.1101/2025.10.10.681671)
Supplement: Supplement 1 [file NIHPP2025.10.10.681671v1-supplement-1.pdf]

bioRxiv preprint doi: https://doi.org/10.1101/2025.10.10.681671; this version posted October 13, 2025. The copyright holder for this preprint (which was not certified by peer review) is the author/funder, who has granted bioRxiv a license to display the preprint in perpetuity. It is made available under aCC-BY 4.0 International license.

5 Supplementary Figures

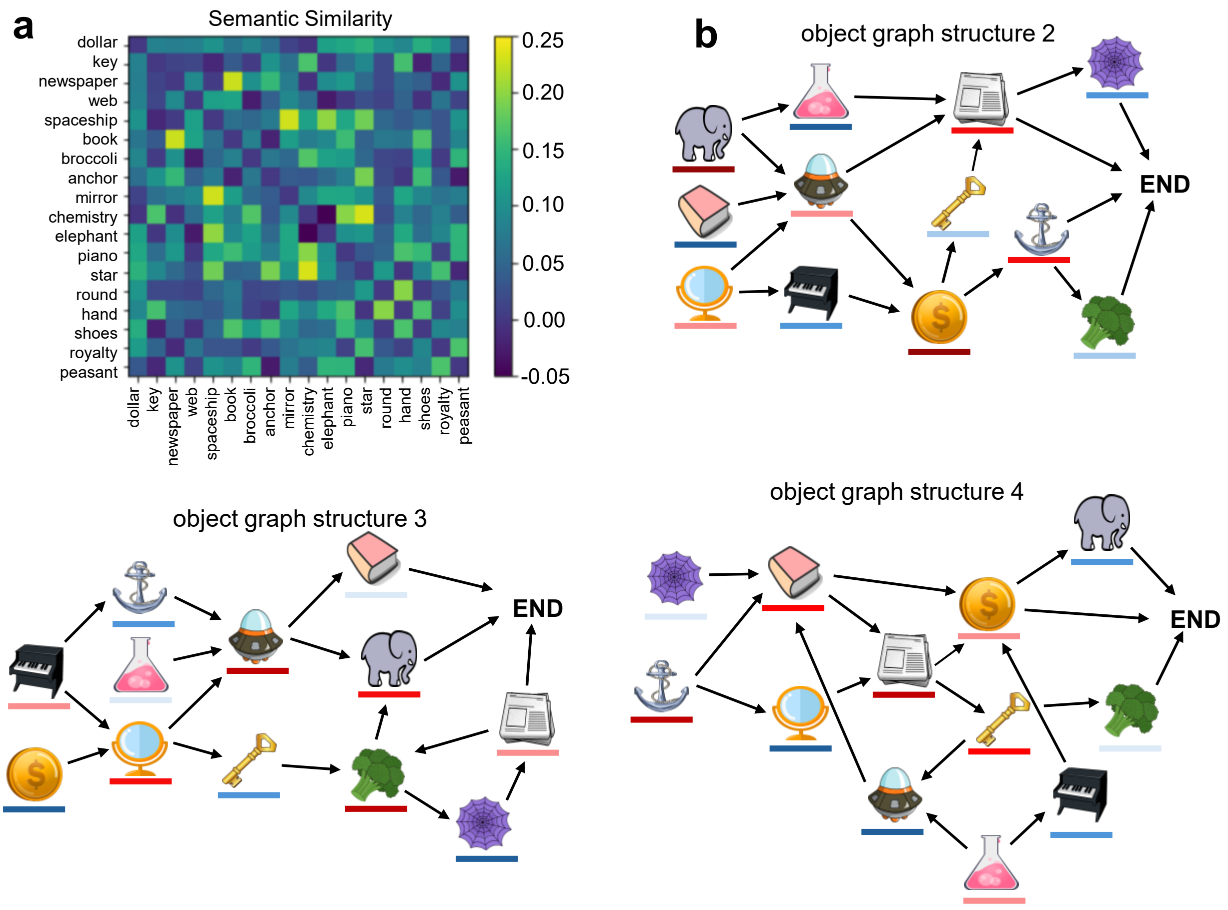

Supplementary Figure 1: Supplementary task design characteristics. a) Semantic similarity between all used stimuli. Stimuli were chosen with the aim of minimizing semantic correlations between them. B) In addition to the graph structure presented in the main manuscript, three additional graph structures were used.

bioRxiv preprint doi: https://doi.org/10.1101/2025.10.10.681671; this version posted October 13, 2025. The copyright holder for this preprint (which was not certified by peer review) is the author/funder, who has granted bioRxiv a license to display the preprint in perpetuity. It is made available under aCC-BY 4.0 International license.

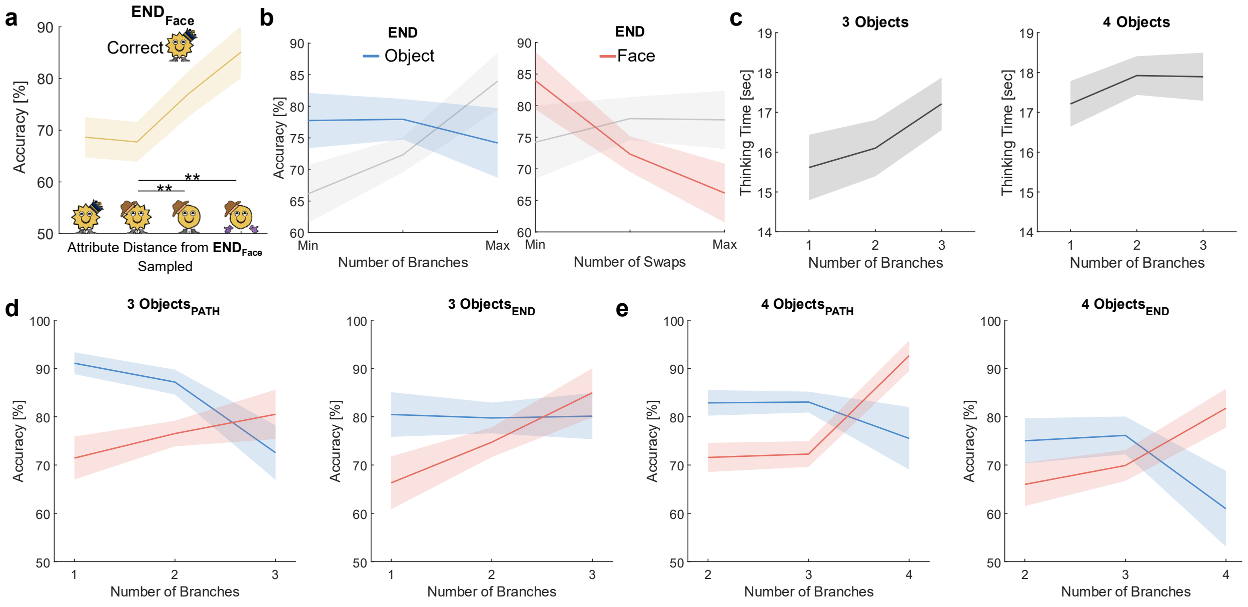

Supplementary Figure 2: Supplementary behavioral results. a) END Face accuracy increased as a function of the attribute distance between the sampled probe and the target END Face. b) Participants' accuracy diverged on END Object vs. END Face questions depending on the number of branches on the correct path. c) The pattern in the planning length decrease was consistent when splitting trials based on program length. de) The pattern in diverging accuracy from panel b and Figure 3I was consistent when splitting trials based on program length and question type (PATH, END).
Thick lines represent mean accuracy across participants, shaded error bar represents SEM across participants.

bioRxiv preprint doi: https://doi.org/10.1101/2025.10.10.681671; this version posted October 13, 2025. The copyright holder for this preprint (which was not certified by peer review) is the author/funder, who has granted bioRxiv a license to display the preprint in perpetuity. It is made available under aCC-BY 4.0 International license.

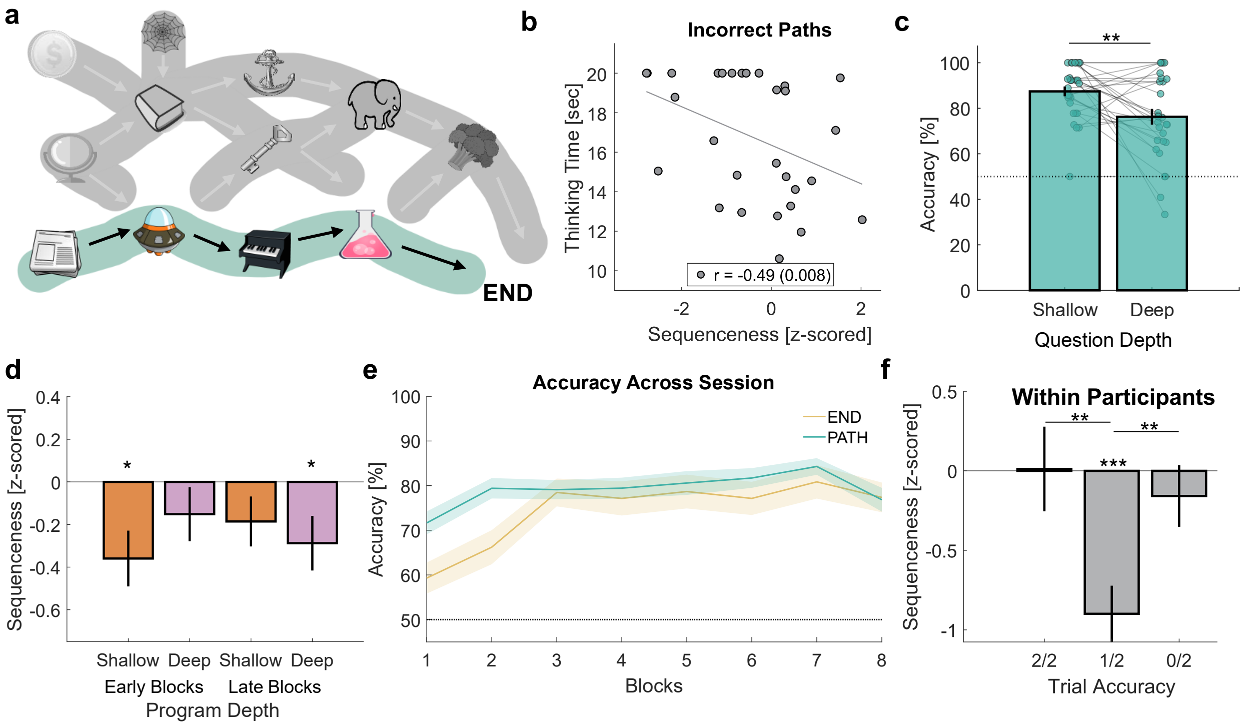

Supplementary Figure 3: Supplementary object replay-related results. a) Correct paths (green shading) refers to the correct path on a trial based on the starting location and face. Incorrect paths (gray shading) refers to all other paths on the graph on the same trial. b) Correlation between incorrect path replay and participants' average thinking time. The correlation remained significant after partialling out differences in overall accuracy (r = -.46, p = .014), correct path replay (r = -.49, p = .009), and did not occur due to a correlation between thinking time and 'replay strength' (p = .99). c) PATH Accuracy decreased as a function of probe question depth (see Methods: Behavioral analysis). d) Replay peak from Figure 5G split based on the first half (Early) and second half (Late) of the session. e) Participants' accuracy improved over the duration of the session. f) Incorrect program path replay peak (60 msec, corrected for multiple comparisons) split based on participants' probe question accuracy. Error bars represent SEM across participants.

bioRxiv preprint doi: https://doi.org/10.1101/2025.10.10.681671; this version posted October 13, 2025. The copyright holder for this preprint (which was not certified by peer review) is the author/funder, who has granted bioRxiv a license to display the preprint in perpetuity. It is made available under aCC-BY 4.0 International license.

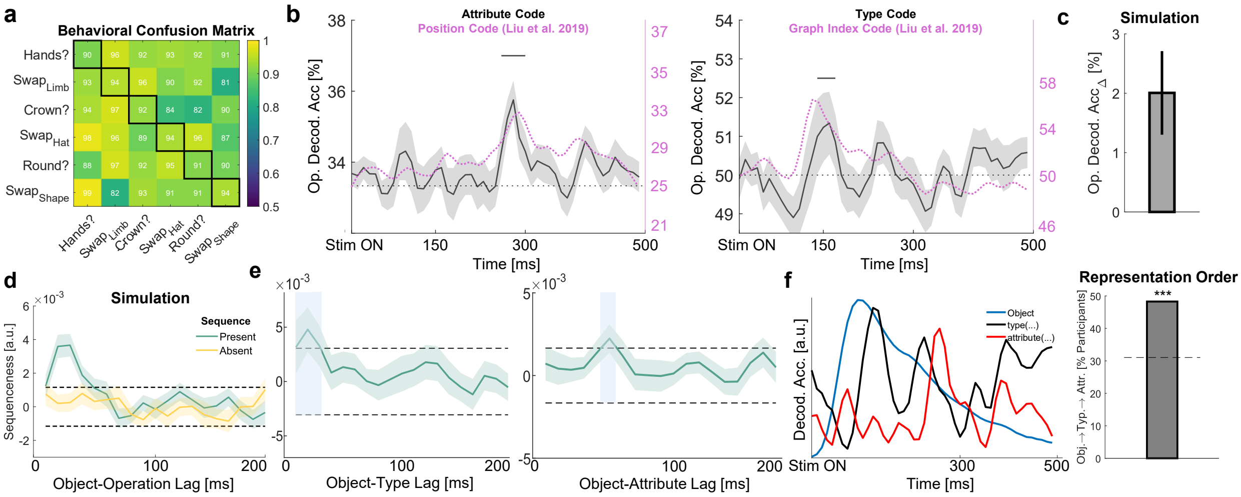

Supplementary Figure 4: Supplementary operation-related results. a) Behavioral confusion matrix on the operation localizer. Numbers along the diagonal represent the probability of correctly accepting the mapping between an object and subsequent operation text label. Numbers off the diagonal represent the probability of correctly rejecting the mapping between image and subsequent operation text label. b) Operation Attribute (left panel) and Type (right panel) decoding across participants in the operation localizer. Shading represents SEM across participants. The black bar denotes significance in cluster-based permutation testing (exceeding the 97.5th percentile of the mass-corrected null). Overlaid are the decoding results of abstract representations from Liu et al.[1]. c) Peak operation decoding accuracy of a decoding model generated using synthetic neural data (n = 20 simulated participants). d) The decoding model from panel c was used to generate synthetic operation reactivations. Applying the TDLM analysis distinguished a condition where Location → Operation sequences existed (green line) from a control condition where they did not (yellow line). e) Sequenceness during the reasoning period for Location → Type (left panel) and Location → Attribute (right panel). Dashed lines indicate the 95th percentile of the null distribution (multiple-comparisons corrected). f) The Location → Type → Attribute representational order implied by the reasoning task sequenceness effect was also present within the operation localizer in 13/29 participants ($p < .001$, Binomial Test, see Methods: Location → Operation sequences). The dashed line represents the 97.5th percentile of a null distribution where representation identity was shuffled.

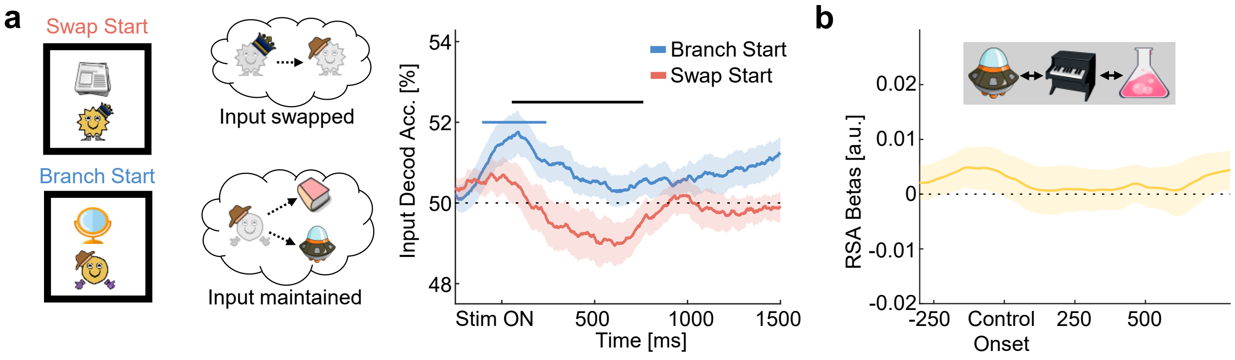

Supplementary Figure 5: Supplementary program execution-related results. a) Left panel: relationship between input representations and the starting locations's operation. Swap Starts predict swapping (Crown → Fedora): decreased decoding of the input representation. Branch Starts predict a maintained input representation to move to the next location. Right panel: At Reasoning Start, the input representation was decoded above chance (blue bar) and was stronger on Branch Start compared to Swap Start trials (black bar). Colored bars represent significance in cluster-based permutation testing (exceeding the 97.5th percentile of the mass-corrected null). b) Control analysis for the result in Figure 7H, locked to onsets of control events (see Methods: Replay-aligned RSA analysis). The thick line represents the mean activity across participants, averaged over several control event initializations (n = 5). Shading represents SEM across participants.
